# Girl predominance in trampoline-related forearm shaft fractures and their increasing incidence since 2000

**DOI:** 10.1186/s12891-023-06241-z

**Published:** 2023-02-28

**Authors:** Markus Stöckell, Ella Pikkarainen, Tytti Pokka, Juha-Jaakko Sinikumpu

**Affiliations:** 1 Department of Pediatric Surgery and Orthopaedics, Oulu University Hospital, Oulu, Finland; 2 Clinical Medicine Research Unit, Oulu Childhood Fracture and Sports Injury Study, and Medical Research Council (MRC), University of Oulu, Oulu, Finland

**Keywords:** Children, Forearm shaft, Fracture, Incidence

## Abstract

**Background:**

There are reports of increasing incidence of forearm shaft fractures in children. Their treatment has been preferably nonoperative but surgical fixation has gained popularity due to elastic stable intramedullary nailing. We aimed to study whether the incidence of pediatric both-bone forearm shaft fractures and their operative care have changed since year 2000. Trampoline injuries, in particular, and their treatment, re-displacement and short-term outcomes were the secondary outcomes of the study.

**Methods:**

A population-based study in the geographic catchment area of Oulu University Hospital district in 20-years of time period (2000 – 2019) was performed. Altogether 481 diaphyseal both-bone forearm fractures in children (< 16 years) were included. Age- and sex-related incidence rates were determined, by using the official numbers of the population-in-risk by Statistics Finland. Trampoline jumping and other types of injury were reviewed, as well as particulars of treatment and outcomes.

**Results:**

The incidence of diaphyseal both-bone forearm fractures increased from 9.4/100 000 in 2000–2001 to 41.7/100 000 in 2018–2019 (*P* < 0.001). Surgical treatment increased respectively (from 8.8/100 000 in 2000–2001 to 35.3/100 000 in 2018–2019, *P* < 0.0001). Trampoline injuries explained one in three (29%) of all fractures; they increased from 0% in 2000–2001 to 36.6% in 2018–2019 (*P* < 0.001). During the last four years of the study (2016–2019), most trampoline-related injuries occurred among girls (61.2%), compared to boys (38.8%) (*P* = 0.031). Trampoline-related injuries comprised 46.9% of all fractures in girls, compared to 26.0% among boys (Diff. 20.8%, 4.7% to 36.1%, *P* = 0.009). The mean age of the patients elevated from 6.4 years (2000–2001) to 8.6 years (2018–2019) (*P* = 0.015). Boys predominated (69.6%) in 2000–2009 but during the last ten years, there was no statistical difference in distribution between the genders (males 54.6%, *P* = 0.11).

**Conclusions:**

During the twenty-year’s of study period, the incidence of pediatric diaphyseal forearm fractures increased fivefold. Trampolining was the most usual single reason for the fractures. More attention should be focused to increase the safety of trampoline jumping, in particular among the girls.

## Background

Approximately one-third of children experience at least one fracture before the age of 17 [1]. Typical injury mechanism for pediatric fractures is fall, while sport related injuries, playground injuries and traffic accidents are usual types of injuries. Forearm shaft fractures comprise up to 15% of all pediatric fractures needing in-hospital treatment [2]. They are challenging to treat, while bone healing is poor as compared to other pediatric fractures and they can result in permanent disability or morbidity [3]. The incidence of forearm shaft fractures has increased four-fold during the first decade of 2000’s [4]. At that time period trampolining caused approximately 25% of all forearm shaft fractures, being the most important single reason for the fracture [4]. In general, trampoline-related injuries in children have become a great challenge, resulting in the high need for preventive interventions [5].

Pediatric forearm shaft fractures are preferable treated with closed reduction and immobilization by a cast [6]. There has been an increasing trend towards surgical intervention, probably as a cause of high (30%) rate of re-reduction during the nonoperative cast treatment [7]. Less follow-up visits and radiographs are needed after operative vs. nonoperative treatment. Several studies indicate that operative care results in good functional, radiological and cosmetical outcomes [8, 9]. Elastic stable intramedullary nailing (ESIN) is the preferred method for osteosynthesis. Postoperative immobilization is seldom needed after ESIN, and surgical wounds are small. Plate and screw fixation is an option in older children [10, 11] because of their higher risk of complications such as nonunion and need of re-reduction [7, 11]. Plate and screw fixation is feasible method in treating meta-diaphyseal forearm fractures, too.

Given that pediatric forearm shaft fractures have increased with high rate during the first decade of the 2000’s, the authors’ aimed to investigate if the incidence of both-bone forearm shaft fractures has persisted in increasing trend. Incidence of diaphyseal forearm fractures in an unselected normal children population (< 16-years’ of age) was the principal aim of the study. Another aims were to investigate trampoline jumping as a background factor of these fractures, and their treatment trends, other injuries, re-displacement and short-term outcomes.

## Methods

This is a population-based cross-section study in a geographical catchment area, where all patients aged less than 16 years with a both-bone diaphyseal middle third forearm fracture during 2000–2019 were included. In total, 481 children and adolescents comprised to the study population. The study center is located at Northern Finland, and it is the only round-the-clock pediatric trauma unit at the region. Regardless on the potential primary treatment at a primary health care unit of few isolated fractures, the clinical and radiographic follow-up of the patients has occurred in the study institution, resulting in that all potential patients were enrolled to the analyses regardless on the place of primary visit. The participation was taken to be inclusive. Information about the patients, injury types, treatment and follow-up were gathered from the hospital data base, while International Classification of Diseases (ICD), version 10, was used for data collecting. All radiographs of the patients were reviewed to confirm the diagnosis. Non-residents were excluded from the study population to avoid bias in the incidence rate. The age-matching population-in-the-risk in the study area was determined by using official descriptive of the population by Statistics Finland. The children population in the study area varied between 84 333 and 88 093 during the study time.

All radiographs associated to the injury were primarily reviewed by a radiologist on-duty and re-reviewed by a clinician not only to confirm the fractures and the eligibility of the patients but also to confirm that only diaphyseal middle third both-bone fractures were included to the data. Displacement and angular deformity of the fractures were analyzed. Fractures with disruption of distal radioulnar joint (Galeazzi fracture and analogous) or radiocapitellar joint (Monteggia fracture and analogous) were excluded. Open fractures were included to the data. Operation reports and post-operative clinical notes were analyzed to find out the recovery of the patients. Treatment was classified as a closed non-invasive treatment (reduction under general anaesthesia and casting or immobilization in-situ) or operative treatment (closed or open reduction and internal fixation by using any method of surgical stabilization).

### Statistics

Incidences were reported per 100 000 age-related persons. Differences between the groups were tested by independent-samples t-test for continuous variables. Chi-squared test was used to test the difference in distributions of the categorical variables. Standardized normal distribution (SND) test was used to analyze the difference of two independent proportions; 2 years’ of groups were used to get satisfactory number of cases, except from genders of the patients with trampoline fractures, when four years of periods were used to get satisfactory groups. Linear trend test was performed to determine the potential change in time. The differences between annual incidence densities during the first two and the last two study years (2000–1 vs. 2018–19) was tested by the chi-square test. Further, descriptive of the study cohort during the first and the last decades were aimed to be presented to present the potential rough trends of the fractures. The estimation in the amount of annual diaphyseal fracture increase was calculated by exponential regression analysis. *P*-value < 0.05 was considered statistically significant, taken that all values were two-tailed, and 95% of confidence intervals were reported when applicable. Statistical analyses were performed using StatsDirect Statistical Software version 3.2.8 and SPSS Statistical Package (version 27.0, IBM Corporation, 2020).

## Results

### Descriptivies of the study cases

**Fig. 1:**
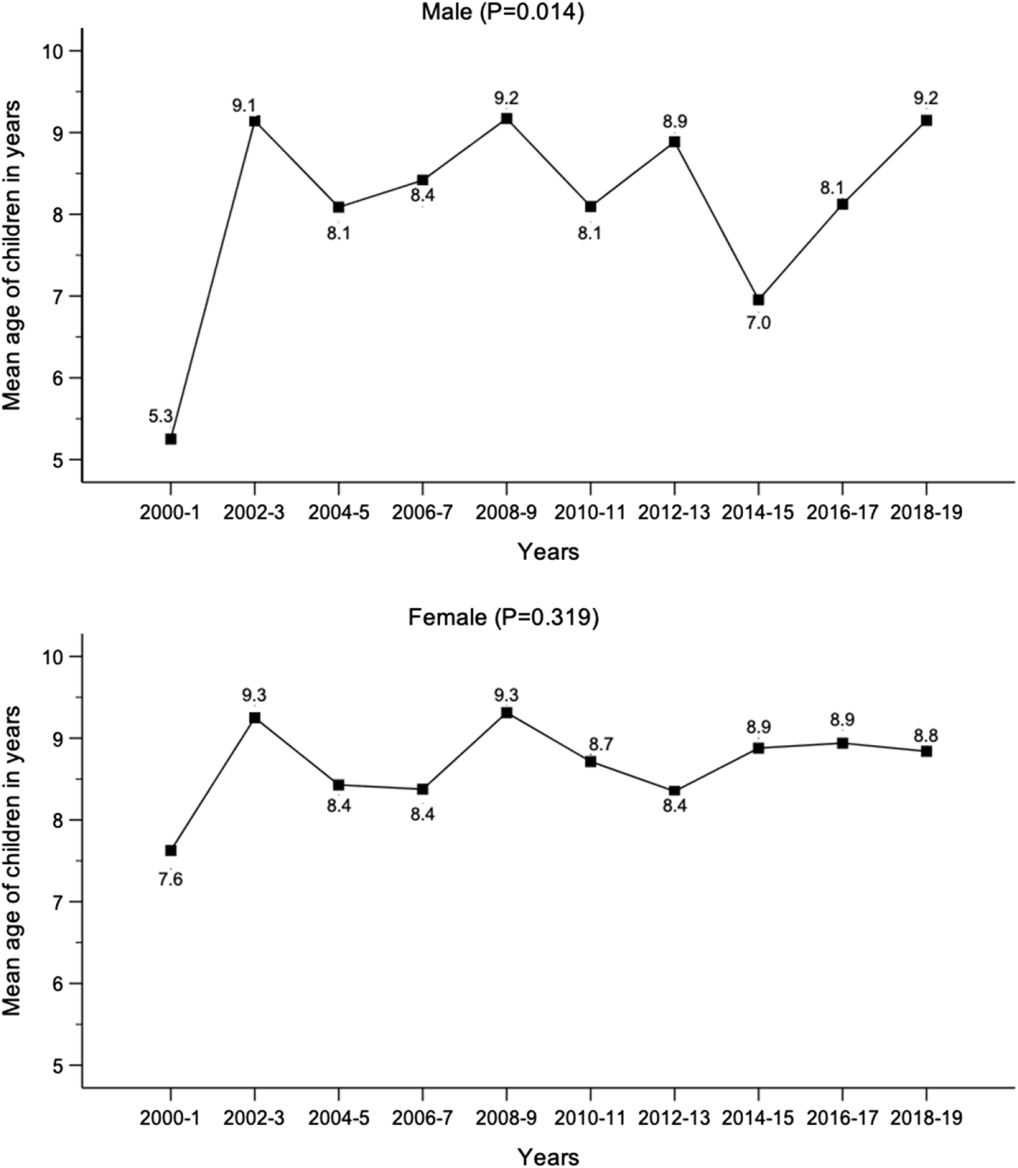
The mean age of pediatric patients with diaphyseal both-bone fracture by gender

There were 481 patients. Altogether 288 (59.9%) were males (*P* < 0.001). The mean age of the boys was 8.4 (Standard deviation (SD) + 3.6) and girls 8.8 (SD + 3.3). The mean age elevated from 6.4 years in 2000–1 (SD + 3.9) to 9.0 in 2018–19 (SD + 3.3) (Fig. 1). The proportion of the girls increased from 30.4% in the first decade (2000–9) to 45.4% in the last decade (2010–2019) (*P* = 0.002) in SND test.

The majority (*n* = 432, 90%) of the fractures were angulated. Shortening was found in 24% (*n* = 114) and multifragmentation in 5% (*n* = 24) of the patients. The rate of open fractures was 8% (*n* = 38) (Table 1).

**Table 1:** Descriptives of pediatric diaphyseal both-bone forearm fractures in the first decade and the second decade of the study period

	2000–2009	2010–2019	All
Number of cases	168	313	481
Gender			
Male	117 (69.6%)	171 (54.6%)	288 (59.9%)
Female	51 (30.4%)	142 (45.4%)	193 (40.1%)
Fracture characteristics			
Open fracture	15 (8.9%)	23 (7.3%)	38 (7.9%)
Comminuted fracture	8 (4.8%)	16 (5.1%)	24 (5.0%)
Angular deformity	137 (81.5%)	295 (94.2%)	432 (89.8%)
Complete displacement	21 (12.5%)	93 (29.7%)	114 (23.7%)

### Incidence

**Fig. 2:**
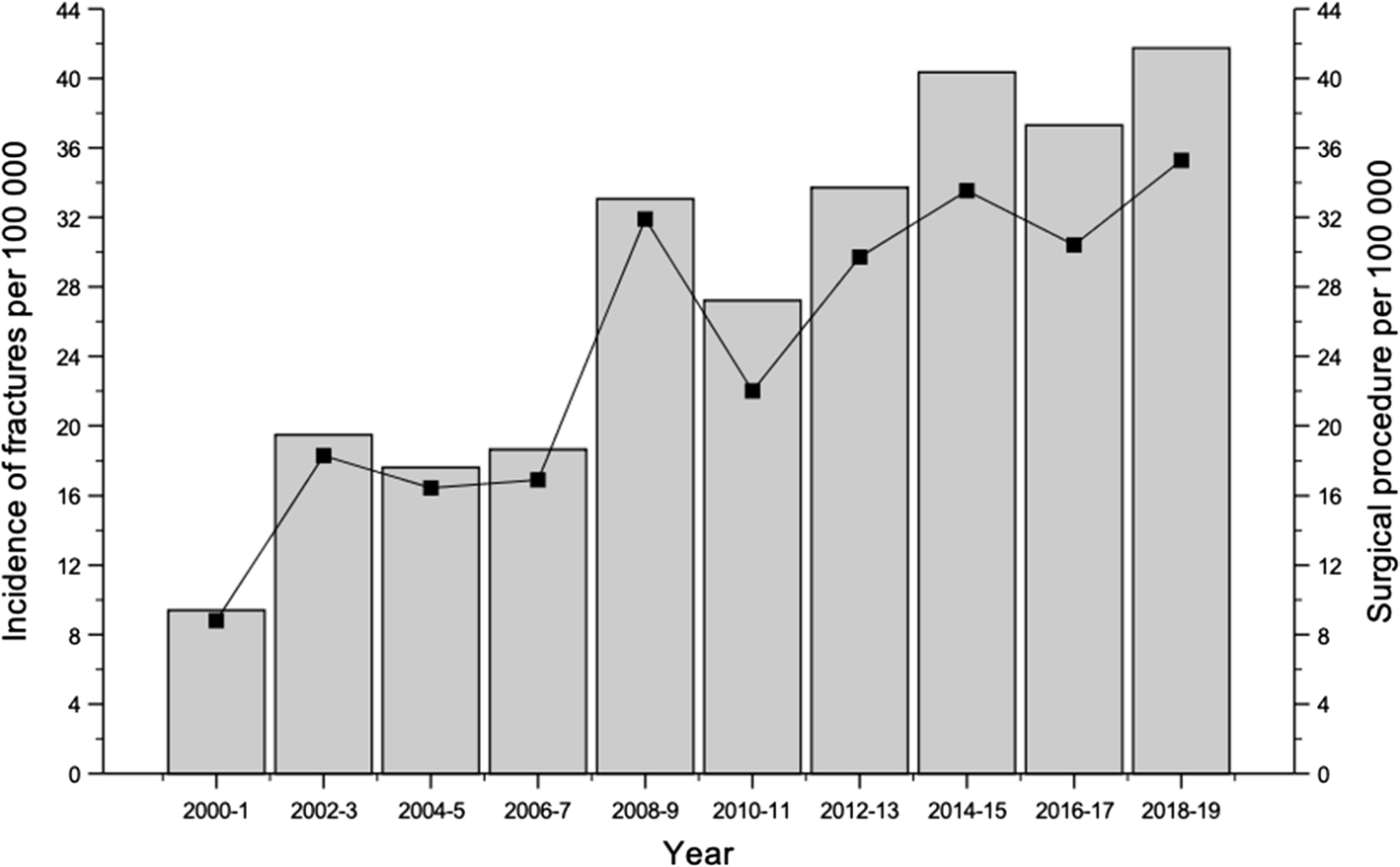
The age-related incidence of pediatric diaphyseal both-bone forearm fractures (bars) and the incidence of surgical procedures for them (line)

The mean annual incidence of forearm shaft fractures was 27.8/100.000 during the entire study period. The incidence increased from 9.4/100 000 in 2000–2001 to 41.7/100 000 in 2018–2019 (Difference (Diff.) 32.3/100.000, 95% CI 22.1/100.000 to 44/100.000%, *P* < 0.001). There was an increase in the incidence of surgical procedures from 8.8/100 000 in 2000–2001 to 35.3/100 000 in 2018–2019 (Diff. 26.5/100.000, 95% CI 17/100.000 to 37.3/100.000, *P* < 0.001) (Fig. 2).

### Trampoline injuries

**Fig. 3:**
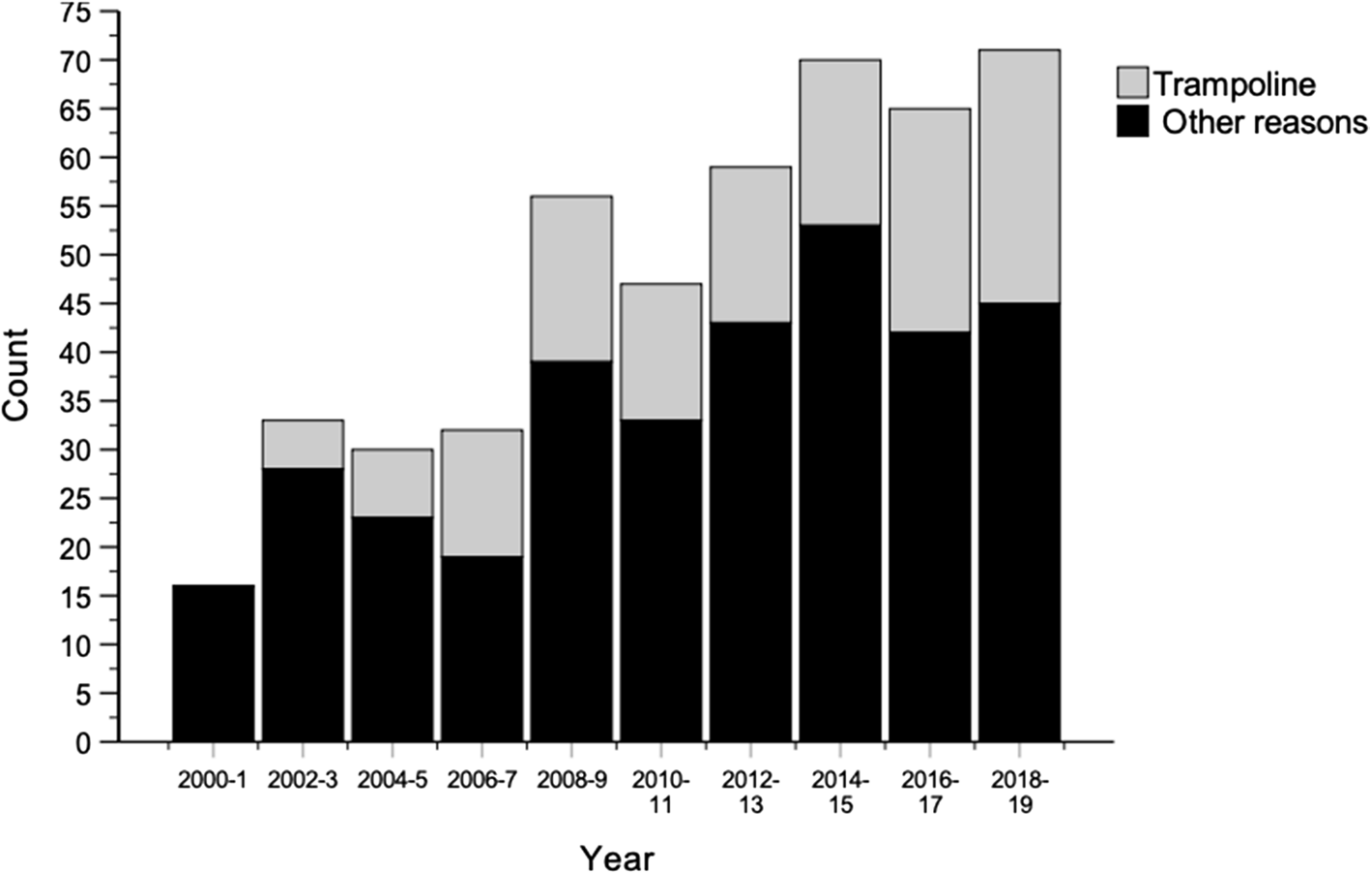
The crude incidence of trampoline related pediatric diaphyseal both-bone forearm fractures and all pediatric diaphyseal both-bone forearm fractures during the study period 2000–2019

Trampoline jumping was associated with every three (29%, *n* = 138) of all fractures. Trampoline injuries increased from 0% in 2000–1 to 36.6% in 2018–2019 (Diff. 36.6%, 95% CI 16.0% to 48.3%, *P* = 001) (Fig. 3). During the last four years of the study (2016–2019), most trampoline-related injuries occurred among girls (61.2%), compared to boys (38.8%) (*P* = 0.031). In total, trampoline-related injuries comprised 46.9% of all fractures in girls, compared to 26.0% among boys (Diff. 20.8%, 4.7% to 36.1%, *P* = 0.009). Other recreational reasons of the fractures are described in the Table 2.

**Table 2:** Injury mechanisms and recreational activities of the children patients, who suffered from diaphyseal both-bone forearm fractures

	2000–1	2002–3	2004–5	2006–7	2008–9	2010–11	2012–13	2014–15	2016–17	2018–19	All^a^
Injury mechanism											
Fall on the same plane	25% (4)	33.3% (11)	20% (6)	18.8% (6)	17.9% (10)	19.1% (9)	18.6% (11)	21.4% (15)	21.5% (14)	29.6% (21)	22.3% (107)
Fall between planes	31.3% (5)	18.2% (6)	13.3% (4)	18.8% (6)	26.8% (15)	29.8% (14)	23.7% (14)	34.3% (24)	24.6% (16)	19.7% (14)	24.6% (118)
Fall on the ice/snow	0% (0)	6.1% (2)	6.7% (2)	3.1% (1)	1.8% (1)	4.3% (2)	6.8% (4)	5.7% (4)	3.1% (2)	1.4% (1)	4% (19)
Fall at the playground	25% (4)	30.3% (10)	33.3% (10)	50% (16)	42.9% (24)	42.6% (20)	37.3% (22)	31.4% (22)	36.9% (24)	43.7% (31)	38.2% (183)
Traffic injury	12.5% (2)	3% (1)	20% (6)	3.1% (1)	3.6% (2)	2.1% (2)	6.8% (4)	1.4% (1)	1.5% (1)	0% (0)	4% (19)
Other injury	6.3% (1)	9.1% (3)	6.7% (2)	6.3% (2)	7.1% (4)	2.1% (1)	6.8% (4)	5.7% (4)	12.3% (8)	5.6% (4)	6.9% (33)
Recreational causes											
Organized sports (soccer, ice hockey, etc.)	18.8% (3)	15.2% (5)	3.3% (1)	9.4% (3)	8.9% (5)	14.9% (7)	11.9% (7)	17.1% (12)	12.3% (8)	18.3% (13)	13.4% (64)
Slalom, cross-country skiing, snowboarding	0% (0)	6.1% (2)	6.7% (2)	3.1% (1)	1.8% (1)	2.1% (1)	3.4% (2)	2.9% (2)	1.5% (1)	0% (0)	2.5% (12)
Bicycling, skateboarding, roller-skating	6.3% (1)	15.2% (5)	20% (6)	0% (0)	8.9% (5)	10.6% (5)	10.2% (6)	5.7% (4)	6.2% (4)	5.6% (4)	8.4% (40)
Trampoline	0% (0)	15.2% (5)	23.3% (7)	40.6% (13)	30.4% (17)	29.8% (14)	27.1% (16)	24.3% (17)	35.4% (23)	36.6% (26)	28.8% (138)
Other playground device, swing, etc	6.3% (1)	9.1% (3)	10% (3)	9.4% (3)	12.5% (7)	17% (8)	15.3% (9)	15.5% (11)	6.2% (4)	11.3% (8)	11.9% (57)
Indoor play	12.5% (2)	9.1% (3)	6.7% (2)	9.4% (3)	8.9% (5)	6.4% (3)	6.8% (4)	7.1% (5)	9.2% (6)	5.6% (4)	7.7% (37)
Motor vehicle	6.3% (1)	0% (0)	0% (0)	9.4% (3)	3.6% (2)	4.3% (2)	3.4% (2)	1.4% (1)	1.5% (1)	0% (0)	2.5% (12)
Falling from ladder, roof, etc	25% (4)	6.1% (2)	6.7% (2)	3.1% (1)	8.9% (5)	8.5% (4)	8.5% (5)	8.6% (6)	10.8% (7)	5.6% (4)	8.4% (40)
Falling when running	25% (4)	24.2% (8)	20% (6)	15.6% (5)	12.5% (7)	6.4% (3)	8.5% (5)	11.4% (8)	12.3% (8)	14.1% (10)	13.4% (64)
Falling from stairs	0% (0)	0% (0)	0% (0)	0% (0)	0% (0)	0% (0)	3.4% (2)	2.9% (2)	3.1% (2)	1.4% (1)	1.5% (7)
Other reason	0% (0)	0% (0)	3.3% (1)	0% (0)	3.6% (2)	0% (0)	1.7% (1)	2.9% (2)	1.5% (1)	1.4% (1)	1.7% (8)

^a^Total number of cases 479 (two cases missing data)

### Surgical treatment

The majority (*N* = 272, 56.5%) of the patients were treated nonoperatively, without surgical fixation, by using cast immobilization with/without previous closed reduction. Nevertheless, there was an increase in surgical care during the study period from 12.5% in 2000–2001 to 39.4% in 2018–2019, (Diff. 26.9%, 1.2% to 42.9%, *P* = 0.027) (Table 3).

**Table 3:** Operative treatment and surgical procedures for the pediatric diaphyseal both-bone forearm fractures

	2000–1	2002–3	2004–5	2006–7	2008–9	2010–11	2012–13	2014–15	2016–17	2018–19	All	*P*-value^**^
Operative activity (% of all patients and number)												
Procedure at OR^a^ (operated)	93.8% (15)	93.9% (31)	93.3% (28)	90.6% (29)	96.5% (55)	80.9% (38)	88.1% (52)	83.1% (59)	81.5% (53)	84.5% (60)	87.3% (420)	
Not admitted to OR (not operated)	6.3% (1)	6.1% (2)	6.7% (2)	9.4% (3)	3.5% (2)	19.1% (9)	11.9% (7)	16.9% (12)	18.5% (12)	15.5% (11)	12.7% (61)	0.007
Surgical procedure (% of all operated and number)												
Closed or open reduction & internal fixation	13.3% (2)	45.2% (14)	35.7% (10)	55.2% (16)	54.5% (30)	63.2% (24)	59.6% (31)	47.5% (28)	49.1% (26)	46.7% (28)	49.8% (209)	
Closed treatment	86.7% (13)	54.8% (17)	64.3% (18)	44.8% (13)	45.5% (25)	36.8% (14)	40.4% (21)	52.5% (31)	50.9% (27)	53.3% (32)	50.2% (211)	0.23

^a^Operating room

^**^Trend test

### Re-operation rate

The need of unplanned operation due to loss of reduction was 26.1% (*N* = 71/272) among the cases who were treated non-invasively primarily. In comparison, the need of re-operation for any reason was found in nine cases (4.3%, *N* = 9/209) among them who were operatively treated primarily (*P* < 0.001).

## Discussion

The main finding of this comprehensive population-based research of childhood forearm shaft fractures was that the incidence of pediatric forearm shaft fractures has increased fivefold during 20-years of period in 2000–2019. The incidence was 8.2 per 100 000 children-in-risk in the beginning of the study, and 40.3 per 100 000 at the end of the study. Trampoline jumping was the most common (29%) single recreational cause of the fractures. Trampoline-related injuries became more frequent and comprised 37% of all fractures at the end of the study (2018–2019). During the last four years of the research, trampoline-injuries happened predominantly in girls (61%), which is an important finding. This finding is opposite to many other childhood fractures, while boys usually predominate in most childhood fractures. The previous literature mainly indicate that boys suffer more usually from forearm fractures, too [6, 12–14]. There is no simple explanation for the predomination of the girls in trampoline-related fractures during the end of the research. However, many reasons for the increasing interest in trampoline jumping by girls can be considered. Trampoline jumping is traditionally associated with gymnastics, which again is traditionally more popular sport among girls than boys. Another explanation may be that indoor trampoline parks have gained popularity among both boys and girls. Indoor trampoline parks with great and powerful high-quality trampolines, compared to conventional backyard trampolines, may predispose children to injuries, especially when playing tricks or somersaults [15]. Furthermore, social media is suggested as a promotor for still more challenging tricks on the trampoline, while video recording of the tricks can incite a child to exceed his/her skills. Given that girls in particular are skilled and active in social media, it is reasonable to hypothesize the use of social media is associated with increased forearm shaft fractures particularly among girls. Nevertheless, that hypothesis needs more research in the future.

A substantial increase in the incidence of both-bone forearm shaft fractures has been reported previously, concerning the beginning of the twenty-first century [4, 13, 14, 16]. However, it has been unclear if such high increase in the incidence could still continue. Our findings suggest that the incidence of forearm shaft fractures continued to increase during the entire 20 years’ of study period, since 2000, albeit the trend became more gentle towards the end of the study. Given that the overall incidence of pediatric fractures has decreased or held stable, such an increasing trend is particularly important [14]. In addition, the forearm shaft fractures in children are one of those that present a real risk of disturbed bone healing and long-term sequelae [3]. Overall complication rate after forearm shaft fractures is 9–22% [17–20], which justify high preventive interventions to avoid these fractures.

In general, there are many factors that have been assumed to explain pediatric fractures. The association between low bone mineral density and increased risk of forearm and wrist fractures has been shown in several studies [21–23]. For instance, low milk consumption and poor calcium intake are associated to low bone mineral density and to a higher risk for fractures [24]. Deficiency of vitamin D was associated with increased risk of forearm fractures as well [22]. Obesity is increasing among children worldwide [25]. Overweight has been reported to be a risk factor for pediatric fractures [22, 26]. Children usually fall against the out-stretched upper extremity, and weight has a solid association with the trauma energy during falling. Increased physical activity has also been associated with increased risk of fractures [12], which is reasonable. Participating in organized sports has increased in the study country recently [27], which have increased the potential injuries for forearm shaft fractures.

In this study, most patients were treated under general anaesthesia in the operating room. The need of surgical intervention increased during the 20 year period. However, the increase was in relation to the increasing number of fractures per se*,* meaning that no change in treatment paradigm was happening. Therefore, the threshold to treat the patients in the operation theatre had been relatively stable during the study time. However, it has been suggested that there is an increasing trend towards invasive treatment such as elastic stable intramedullary nailing in the treatment of both-bone forearm shaft fractures [17, 28, 29]. This is opposite to the previous reports which have stated that surgical fixation has become more popular as an alternative to nonoperative care, due to high occurrence rate of redisplacement, especially in older children [6, 30]. However, in our study, increase in the surgical treatment of forearm shaft fractures was found but it was depending on the increased frequency of the fractures and not on the change in treatment strategy from nonoperative to operative care. If surgical stabilization in needed, ESIN is a preferred method. It is minimal invasive technique, and the surgical wounds are far from the fracture site, which will enhance the healing process. In addition, it produces decent stability [10] resulting in good functional and cosmetical outcomes [31–33], without routineous postoperative cast immobilization [34]. However, our current research suggests that nonoperative treatment had remained as the preferred option over surgical treatment, and principally, osteosynthesis would be reserved for older children, beyond the age of 10 or more, and to the cases, whose remodeling potential is low [30, 35, 36].

There are several strengths in this study. The research was performed in a catchment area of about 88.000 children and the enrolment can be taken as inclusive. There are few private clinics in the area that may have treated some isolated pediatric forearm shaft fractures, but the number of these potential patients were considered to be infinitesimally small; the study institution is the only round-the-clock pediatric trauma unit, familiarized with ESIN, and the unit where follow-up of the children fracture patients is performed. Non-residents were excluded from the data and the official numbers of children-in-risk was precise and certain. As another strength, radiographs were obtained from all patients, and close radiographic particulars of the cases were reviewed. As limitations, this is retrospective study and the decision to operate or not has not been randomized. The decision of treatment was made by a treating surgeon-on-call individually for every patient. Another weakness is that at least two-years’ of time periods were needed to get satisfactory groups for analyses. Differences in genders (girls vs boys) in a subgroup of trampoline fractures only was analyzed in 4-years’ of time periods, to get satisfactory groups for analyses. However, classification of the cases in two to four years’ of study groups worked properly, answering to the study aims with satisfactory accuracy.

## Conclusion

As a conclusion, we found fivefold increase in the incidence of pediatric diaphyseal both-bone forearm fractures during the last twenty years (2000–2019). Trampoline-related fractures increased, comprising 37% of all forearm shaft fractures at the end of the study. Girls predominated in trampoline-related fractures, which is new finding and justify further research.

## Data Availability

The datasets used and/or analyzed during the current study are available from the corresponding author on reasonable request.
